# Exploring the Biological Pathways of Siderophores and Their Multidisciplinary Applications: A Comprehensive Review

**DOI:** 10.3390/molecules29102318

**Published:** 2024-05-15

**Authors:** Benkang Xie, Xinpei Wei, Chu Wan, Wei Zhao, Renfeng Song, Shuquan Xin, Kai Song

**Affiliations:** School of Life Science, Changchun Normal University, Changchun 130032, China; kkangyyds@163.com (B.X.); weixinpei@yeah.net (X.W.); wanchuccsf@163.com (C.W.); weiweizcn@163.com (W.Z.); songsongrenfeng@163.com (R.S.)

**Keywords:** siderophores, biosynthesis, secretion, iron transport, environmental applications

## Abstract

Siderophores are a class of small molecules renowned for their high iron binding capacity, essential for all life forms requiring iron. This article provides a detailed review of the diverse classifications, and biosynthetic pathways of siderophores, with a particular emphasis on siderophores synthesized via nonribosomal peptide synthetase (NRPS) and non-NRPS pathways. We further explore the secretion mechanisms of siderophores in microbes and plants, and their role in regulating bioavailable iron levels. Beyond biological functions, the applications of siderophores in medicine, agriculture, and environmental sciences are extensively discussed. These applications include biological pest control, disease treatment, ecological pollution remediation, and heavy metal ion removal. Through a comprehensive analysis of the chemical properties and biological activities of siderophores, this paper demonstrates their wide prospects in scientific research and practical applications, while also highlighting current research gaps and potential future directions.

## 1. Introduction

Iron is essential for the growth and development of all living organisms [1], serving as a cofactor for crucial enzymes in oxidative metabolism and as a significant component of oxygen transport proteins [2]. Although iron is abundant in nature, it predominantly exists in an oxidized Fe^3+^ state, forming stable iron oxide compounds. Consequently, the concentration of free iron available for microbial use in natural environments is deficient, around 1 × 10^−18^ mol/L, compared to the 1 × 10^−6^ mol/L required by most microbes [3]. Siderophore is a kind of low-molecular-weight water-soluble organic compound that binds explicitly trivalent iron ions in a low-iron environment, and is mainly secreted by microorganisms and some plants (such as Gramineae). It has strong affinity for trivalent iron ions and can form trivalent iron chelates [4]. Organisms have developed efficient iron transport mechanisms, including siderophores, to cope with iron-limited conditions. These tiny molecular secretions play a vital role in capturing and transporting external iron ions [5].

Recent studies highlight the versatility of siderophores, showcasing their potential in fields such as drug development [6,7,8], environmental management [9,10,11], and agriculture [12,13,14]. Research efforts also focus on extracting and identifying siderophores [15,16]. Currently, 649 functions have been defined [17], while other functions are still under investigation [18].

Siderophores are not merely crucial biomolecules; they also hold significant promise in the life sciences, medicine, and chemistry sectors. Despite their pivotal role, comprehensive reviews of siderophore research are sparse. This paper thoroughly examines and analyzes the diverse classification, biosynthetic pathways, and secretion mechanisms of siderophores in microorganisms and plants, along with their applications across various fields. It aims to provide a comprehensive domain of this domain’s current research status, development trends, and pressing issues.

Moreover, this paper summarizes existing research findings and delves into future research directions and potential developmental trajectories, offering a valuable reference for researchers in related disciplines to foster further advancements and innovations in siderophore research.

## 2. Results

### 2.1. Classification of Siderophores

Siderophores are classified into three primary categories based on their chelating groups: catecholate-type [19,20], hydroxamate-type [21,22], and carboxylate-type [23,24]. In addition to the above types, some siderophores can also be classified as mixed siderophores (Table 1). Among these, hydroxamate-type siderophores, which are the most prevalent in nature, are produced by both bacteria and fungi. In bacteria, these siderophores consist of acylated and hydroxylated alkylamines [25], whereas in fungi, they are made of hydroxylated and alkylated ornithine [26]. Their iron binding constants range from 10^22^ to 10^32^ L/mol [27]. Exclusively found in bacteria, catecholate-type siderophores like enterobactin from *Escherichia coli* and salmochelin from *Klebsiella pneumoniae* are known for their lipophilicity, high affinity for iron, and resistance to pH changes, with binding constants reaching up to 10^52^ L/mol [28]. Carboxylate-type siderophores, although less common, are produced by specific bacteria like *Alfalfa rhizobia*, which use carboxyl and hydroxyl groups to bind with iron.

### 2.2. Synthesis of Siderophores

Siderophores are synthesized through two principal mechanisms: one involves the nonribosomal peptide synthetase (NRPS) pathway, and the other is the nonribosomal-independent synthesis (NIS) pathway.

#### 2.2.1. NRPS Pathway Synthesis of Siderophores

**Figure 1:**
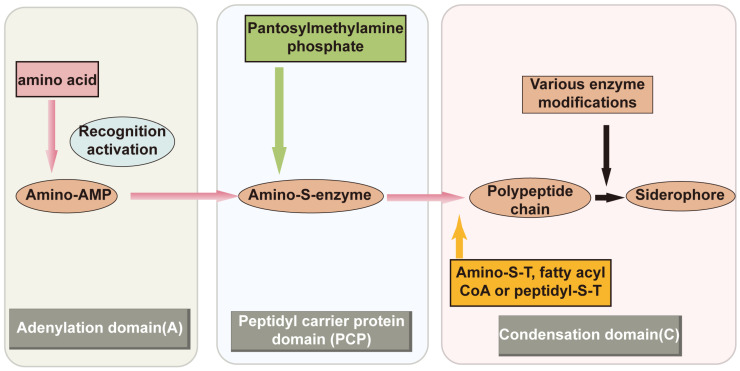
Process of synthesizing siderophore through the NRPS pathway. The diagram features three large boxes, aligned from left to right, representing the adenylate domain, peptidyl carrier protein domain, and condensation domain. The arrow indicates the direction of the synthesis process, while the oval and square figures denote the various substances involved.

The NRPS pathway is the primary mechanism for producing most siderophores and involves complex, multi-modular enzymes that perform a series of coordinated steps to synthesize nonribosomal peptides. Each NRPS module typically consists of three key domains: the adenylation (A) domain, the peptidyl carrier protein (PCP) domain, and the condensation (C) domain. These domains work in tandem to accurately synthesize siderophores (Figure 1) [31].

The synthesis begins when the A domain recognizes and selects a specific amino acid substrate, activating it into an aminoacyl-AMP intermediate. This intermediate is then transferred to the adjacent PCP domain, which attaches to a phosphopantetheinyl thiol, forming an aminoacyl-S-enzyme intermediate. This intermediate is moved to the C domain, where it undergoes condensation with either the upstream aminoacyl-S-T domain complex, acyl-CoA, or peptidyl-S-T domain complex, thus forming a peptide bond and extending the peptide chain. This sequential functioning of NRPS modules assembles the amino acids into a siderophore peptide chain with a defined structure, ensuring the structural and functional integrity of the final product.

Some siderophores also include fatty acid chains at the N-terminus, creating nonribosomal lipopeptides. Liang et al. [32] found liposiderin produced by Pseudomonas putida. The incorporation of these fatty acids is usually catalyzed by the initiating condensation (Cs) domain. For these fatty acids to participate in biosynthesis, they must first be activated into coenzyme A or acyl carrier protein forms, enabling their integration into the nonribosomal lipopeptide structure.

#### 2.2.2. Synthesis of Siderophores Independent of the NRPS Pathway (NIS)

Aside from the well-documented NRPS pathway, siderophores can also be synthesized through the nonribosomal-independent synthesis (NIS) pathway [33]. This mechanism was first recognized in the production of aerobactin by *E. coli*. The NIS pathway operates through the concerted efforts of several enzymes, utilizing dicarboxylic acids and either diamines or amino alcohols as the foundational building blocks. These components are linked via specific chemical bonds to produce either hydroxamate-type or carboxylate-type siderophores.

**Figure 2:**
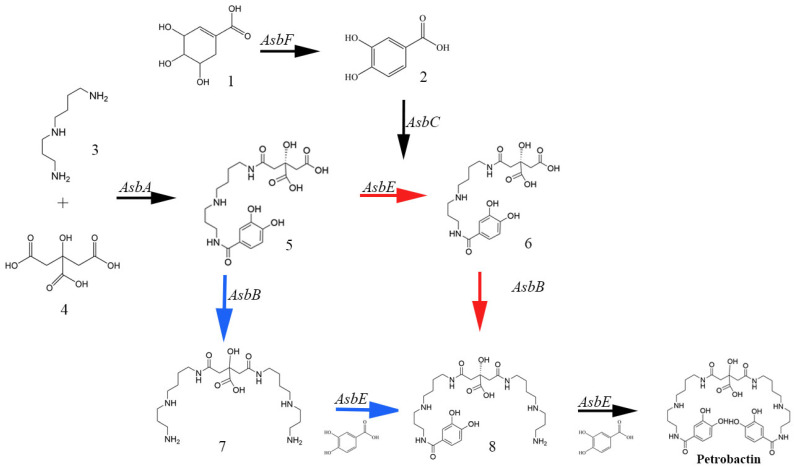
Process of producing petrobactin by *Bacillus anthracis*. Precursors shikimic acid, spermidine, and citric acid finally synthesized siderophore petrobactin through different routes and under the catalysis of different enzymes.

Although research on the NIS pathway is still emerging, current understanding categorizes NIS synthases into three classes based on their substrate specificity: Class A, which targets citric acid chiral root groups; Class B, which focuses on α-ketoglutaric acid root groups; and Class C, which works with esterified or amidated derivatives of carboxylic acids [34]. A detailed example of the NIS pathway can be seen in the synthesis of petrobactin by *Bacillus anthracis*. Initially, shikimic acid is transformed into a 3,4-DHB ligand by the dehydration enzyme AsbF, it was transferred to the skeleton of citroyl spermidine by the synthetase AsbE. This is followed by Class A NIS synthase AsbA, which catalyzes the formation of (3S)-N8-citroyl spermidine by linking spermidine and citric acid. From here, the pathway diverges into two branches. One pathway continues with AsbE synthase, which combines 3,4-DHB with N8-citroyl spermidine (as shown by the red path in the figure). The other pathway proceeds through Class C NIS synthase AsbB, producing citroyl bis-spermidine (As shown by the blue path in the figure). The intermediates from these pathways are then joined either by AsbB or AsbE. Finally, the synthesis culminates with AsbE adding the second 3,4-DHB ligand to complete the biosynthesis of petrobactin (Figure 2) [31].

With the progress in bioinformatics and the expansion of genomic sequencing, researchers have been able to identify a broader array of genes that encode NIS synthases. Gene clusters containing these enzymes have been discovered in over 40 different types of microorganisms, including plant pathogens, animal pathogens, saprophytic fungi, and the symbionts of both plants and animals. The initial identification of an NIS synthase, DesD, occurred in Streptomyces. This enzyme is crucial for the production of desferrioxamine, a siderophore that significantly aids in the growth and differentiation of the organism [35,36]. The discovery of DesD and similar enzymes underscores the importance of the NIS pathway in siderophore synthesis. It highlights the critical role these pathways play in microbial growth and their interactions with host organisms, demonstrating the interconnected nature of microbial ecology and host relationships.

### 2.3. Sources of Siderophore Secretion

#### 2.3.1. Plants

In iron-deficient environments, plants enhance their iron uptake and utilization by producing siderophores (Table 2), crucial for maintaining normal growth under these challenging conditions. Members of the grass family, especially, have been found to produce siderophores. Research led by Nakib et al. has demonstrated that cultivated barley and wild barley exhibit significant differences in siderophore secretion under varying iron concentrations, indicating intraspecific adaptive variations [37]. This capability helps grass family plants manage iron deficiency effectively, improving their iron acquisition and utilization. However, reports of siderophore synthesis and secretion by other plants under iron-deficient conditions are relatively scarce, suggesting a potential area for future research.

#### 2.3.2. Microorganisms

Microorganisms, including some fungi and bacteria, secrete siderophores, i.e., low-molecular-weight organic compounds with a high affinity for iron ions that form stable chelates with Fe^3+^. These compounds are vital for microorganisms in iron-poor environments (as shown in Table 3). For instance, research by Sullivan et al. uncovered microorganisms in African dust that produce high levels of siderophores, providing insight into microbial adaptation in iron-deficient conditions [42]. Additionally, Perez-Miranda et al. and colleagues have developed an efficient, non-toxic method for determining siderophore production in microorganisms cultured on solid media, aiding the study of siderophore production traits within microbial populations [43].

### 2.4. Secretion and Mechanism of Action of Siderophores

**Figure 3:**
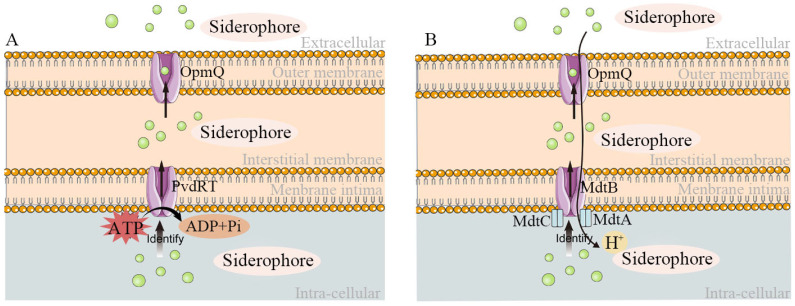
Schematic diagram of the secretion process of siderophores. (**A**) ABC-type efflux system. After being identified, the siderophores in the cells are excluded from the cells by crossing the membrane on the basis of energy provided by ATP. (**B**) RND efflux system, in which siderophores in cells are excreted across the membrane after the recognition of special proteins, and the energy of this process comes from the dynamic potential of protons.

Siderophores, once synthesized, are typically secreted from the cell through specific pathways. In Gram-negative bacteria, this involves ATP-binding cassette (ABC) and resistance–nodulation–cell division (RND) efflux systems [49]. For example, *Pseudomonas aeruginosa* utilizes the trimeric efflux system PvdRT-OpmQ (ABC-type) to export newly synthesized siderophores, while *Pseudomonas putida KT2440* uses both PvdRT-OpmQ and the trimeric efflux system MdtABC-OpmB (RND-type) for this purpose [50,51]. The process in the ABC system starts with the PvdRT-OpmQ complex recognizing siderophores inside the cell. Energy is then expended to transport the siderophore across the inner membrane to the periplasm. Once in the periplasm, it moves through the OpmQ channel protein to exit through the outer membrane. Conversely, in the RND system, siderophores are recognized by the MdtA and MdtC proteins and transported externally through a channel formed by MdtB and OpmQ proteins, driven by the proton motive force of the cell (Figure 3).

**Figure 4:**
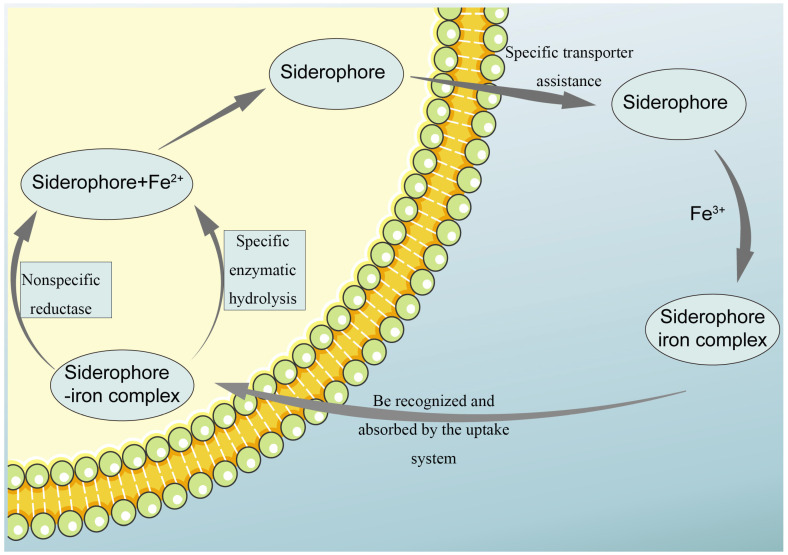
Schematic diagram of the action mechanism of siderophores. After being excreted from the cell, the siderophores in the cell combine with Fe^3+^ in the surrounding environment to form chelates. After being recognized by the cell, the chelates are reabsorbed into the cell, and iron is released in the form of Fe^2+^ in two ways for the cell to use.

As illustrated in Figure 4, the action mechanism of siderophores involves their secretion into the extracellular environment, where they bind to iron ions. This complex formation is facilitated by specific transport proteins that transfer siderophores from inside the cell to the outside, creating soluble complexes with iron ions. These complexes are then recognized and absorbed by the microbial uptake system [52]. Once inside the cell, iron ions are dissociated from the siderophores through two primary methods [53]. The most common method involves non-specific siderophore reductases that reduce ferric ions to ferrous ions, a process essential for iron absorption in plants. Flavin reductases play a pivotal role in this reduction. Alternatively, iron release can occur through the specific enzymatic hydrolysis of siderophores. This process alters the structure of the siderophores, weakening their bond with the iron ions and thereby facilitating iron release [54]. The freed iron ions are then available for various biosynthetic processes within the cell, making this pathway a crucial mechanism for biological iron acquisition.

### 2.5. Functions of Siderophores

#### 2.5.1. Sustaining Normal Biological Activities

Iron is a critical micronutrient essential for the survival and growth of living organisms, playing a pivotal role in various biological processes. In nature, most iron exists as Fe^3+^, a form that cannot be directly absorbed by plant roots, leading to conditions such as iron-deficiency chlorosis, particularly in trees. This deficiency poses a severe global challenge [55]. Iron’s importance extends to its role in hemoglobin within animal blood, where deficiency represents one of the most significant nutritional shortfalls worldwide. Consequently, research focused on ameliorating iron deficiency in crops is crucial not only for boosting agricultural production, but also for its broader implications for human health.

In recent years, with the deepening of the research on siderophores, more and more attention has been paid to siderophores secreted by microorganisms (Table 4 shows that the data come from the WOS core database). Gao et al. [56] and Ghavami et al. [57] found that iron-secreting microorganisms can increase the iron content of plants, as well as promote the absorption of other elements such as zinc and phosphorus [58].

#### 2.5.2. Biological Control

Siderophores exhibit natural biocontrol properties by inhibiting the growth and reproduction of pathogens through iron ion sequestration, effectively suppressing disease propagation. Many bacteria and fungi can secrete one or several types of siderophores, which are critical in managing bacterial and fungal pathogens. The underlying principle of using siderophores for biocontrol is their competition for iron resources, which starves pathogens of the iron necessary for their growth, leading to inhibition or death [59]. This concept was first highlighted by Kloepper et al., who demonstrated that siderophores produced by *fluorescent pseudomonads* isolated from potato skins or roots could suppress *Erwinia* carotovora, a pathogen responsible for seed rot [60]. Further research by Schiessl et al., within the *Pseudomonas aeruginosa* system, showed that the mechanisms involving siderophore-mediated low iron solubility significantly influence competition for iron resources [61]. Much of the research in biocontrol focuses on pyoverdines secreted by the *fluorescent pseudomonad* group due to their strong antibacterial properties against various pathogens [62].

#### 2.5.3. Environmental Protection

The role of siderophores extends to environmental protection, where they facilitate iron transport through specific receptor proteins. This unique feature has potential applications in fields like drug targeting using siderophores. The “Trojan horse” strategy exemplifies this, where siderophores are used as carriers to deliver drugs specifically to pathogenic microbes. This method enhances the precision of pathogen targeting and reduces reliance on pesticides, contributing to safer and more targeted therapeutic interventions [63].

#### 2.5.4. Disease Treatment

Siderophores have potential applications as antibiotics. Negash et al. highlighted recent developments in siderophore–antibiotic conjugate drugs [64]. Furthermore, Patel et al. explored using siderophores as a basis for designing drugs that inhibit *Mycobacterium tuberculosis* [65]. These strategies leverage the unique iron binding capabilities of siderophores for disease treatment, and are emerging as significant areas of future research.

#### 2.5.5. Remediation of Hydrocarbon Pollution

Siderophores also play a crucial role in the remediation of hydrocarbon pollution in marine environments. They indirectly aid in the biodegradation of hydrocarbons by enhancing microbial iron acquisition under iron-limited conditions. Gauglitz et al. [66] isolated a marine arcobacter from the Gulf of Mexico after an oil spill that produces an amphipathic siderophore, effectively promoting the degradation of petroleum hydrocarbons. Additionally, some studies have reported that siderophores can enhance the degradation of nuclear waste [67].

#### 2.5.6. Remediation of Heavy Metal Pollution

Heavy metal contamination from industries like manufacturing, nuclear power, and mining is a severe environmental issue [68]. Siderophores can solubilize various metals, such as copper, chromium, and lead, particularly in copper-contaminated environments where the abundance of siderophore-producing microbes and the total quantity of siderophores increase significantly [69]. The use of siderophores in metal bioremediation is noted for its low cost, high efficiency, and environmentally friendly approach. For instance, Neubauer et al. demonstrated that desferrioxamine B can chelate Co^3+^ more effectively than Fe^3+^ in alkaline conditions [70].

Two primary approaches are used to treat heavy metals chelated by siderophores: microbes like *mangrove fungi* and *Fusarium solani* can accumulate copper and zinc in their biomass [71], and *Bacillus subtilis* can specifically accumulate cadmium, offering insights into the microbial mechanisms of heavy metal enrichment [72]. Additionally, plants can absorb these metal chelates; for example, siderophores from *Pseudomonas aeruginosa* and *Pseudomonas fluorescens* enhance maize’s uptake of chromium and lead [73]. Siderophores can also mitigate heavy metal toxicity in plants, as shown by hydroxamate-type siderophores from Aspergillus species reducing arsenic poisoning in wheat [74], and dihydroxamate-type siderophores from *cyanobacteria* decreasing cadmium poisoning risk in rice [75].

#### 2.5.7. Additional Functions

Siderophores can significantly reduce the use of chemical agents in the bleaching of sulfate pulp in the paper industry by 70% [76]. This reduction occurs as siderophores convert Fe^3+^ to Fe^2+^, which reacts with H_2_O_2_ to produce free radicals that decompose cellulose, hemicellulose, and lignocellulose, thereby achieving the bleaching effect [77,78]. Furthermore, some siderophores may exhibit pathogenic properties. While siderophores themselves are not proven toxic, the ability to produce siderophores is a common trait among all pathogenic Bacillus species. Notably, Bacillus strains known to be potentially pathogenic to humans and animals produce the siderophore petrobactin. Anthrax Bacillus lacking siderophore-transport-related genes showed reduced virulence in mouse models [79]. Additionally, siderophores can act as “weapons” against host cells; Wilson et al. found that Pseudomonas syringae uses the siderophore pyoverdine to sequester iron from host cell proteins, such as lactoferrin, leading to host cell death [28].

## 3. Research Trends and Hotspots in the Field of Siderophore Secretion

### 3.1. Annual Publication Volume

**Figure 5:**
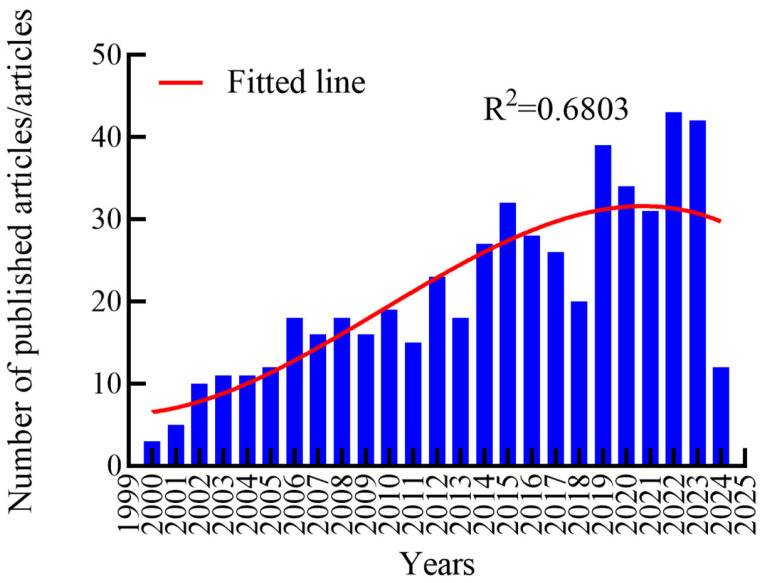
The total amount and trend of English articles published from 2000 to 2024. The red line represents the fitting line of the map, and the blue band represents the number of articles published each year.

Figure 5 shows the number of articles published in the Web of Science core database from 1 January 2000 to 31 March 2024 related to siderophore secretion. The number of documents in this field shows an overall growth trend from the overall trend. The average annual publication rate from 2014 to 2023 was 32.2 papers, a significant increase from the average of 16.6 papers from 2004 to 2013. This surge indicates heightened attention toward the study of siderophores, spurred by their growing recognition for practical applications, such as in bioremediation and for their antimicrobial properties [80,81]. The data reveal an initial publication count of three papers by 2000, with 2019 recording the highest annual growth rate at 57.1%. This peak year marks a notable expansion of siderophore research from its traditional domains of biochemistry and molecular biology into broader fields like microbiology [27]. From 2006 to 2023, the publication volumes show fluctuations but maintain an average of 25.8 papers per year, suggesting a mature period of consistent research focus on siderophore types [27], synthesis regulation mechanisms [82], functions [83], and applications [84].

The increasing interest is also reflected in significant research outputs, such as the work by Destoumieux-Garzon et al., which demonstrated how siderophore modifications could enhance the antimicrobial activity of microcins in *E. coli* [85]. Similarly, Patel et al. explored the influence of siderophores on the survival and growth of *Mycobacterium tuberculosis* [65]. These studies highlight the potential of siderophores in medical and ecological applications, including research by Negash et al. on siderophore–antibiotic conjugates to combat antibiotic resistance [64], and Leventhal et al. work on the role of siderophores in chelating iron within marine environments for environmental remediation [86]. Despite a stable interest in the field, there were noticeable dips in publication volumes in 2013 and 2018, followed by subsequent recoveries. The year 2013 was particularly prolific, with high-quality publications that explored new research directions, such as Condon, BJ’s study on the virulence of siderophores in maize pathogens [87] and Olejnickova et al.’s confirmation of the bioenvironment’s impact on the toxicity of siderophores in *Pseudomonas aeruginosa* biofilms [88]. Another example is Silva-Baila et al. By studying the iron acquisition mechanism of Paraspora, Silva-Baila et al. found that siderophore production is related to fungi’s pathogenicity [89]. According to statistics, in 2013 alone, articles on the field of siderophore secretion have been cited 1106 times, accounting for 9.7% of the total cited times in this database. This remarkably high number of citations just reflects the importance and influence of the research results in 2013. In 2018, the research on siderophores has approached maturity, and the key words have not been updated, indicating that no new progress and new discoveries have been made in the research on siderophores in that year. However, 2019 saw emerging research areas like rhizosphere and plant growth promotion, with studies like Mucha et al. on the effects of siderophores on the organelles of Scots pine roots [90] and Chen et al. on the production of endophytic siderophores in peanuts, which were linked with enhanced plant growth [91]. These studies fueled a renewed wave of research interest, underscoring the dynamic nature of siderophore research and its evolving implications for both the scientific community and practical applications.

### 3.2. Analysis of Research Hotspots

**Figure 6:**
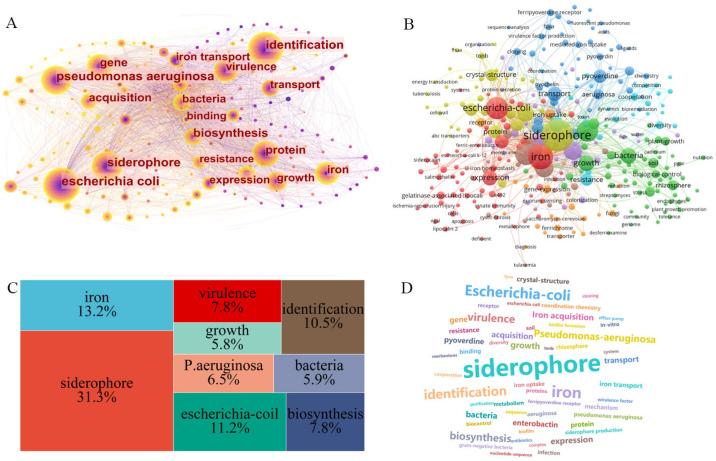
Key words in the field of iron carrier secretion. (**A**) Keyword clustering view made by Cite Space (V.6.2.R4) software. The color change from blue to yellow represents the year when the keyword was studied from 2000 to 2024, and the size of the node represents the importance of the keyword. The bigger the node, the more important the keyword is. (**B**) Keyword pop-up view made by VOS viewer (V1.6.20) software, and the size of nodes represents the importance of keywords. The bigger the node, the more important the keyword is. The color of nodes represents the relationship between different keywords, and the keywords with the same color are more closely related. (**C**) Tree diagram of the keyword matrix made for the micro-letter webpage, and the larger the area occupied by keywords, the more critical it is. (**D**) Keyword cloud made by the chart show webpage, and the bigger the keyword font size, the more important it is.

Using sophisticated bibliometric tools like Cite Space and VOS viewer, a comprehensive cluster analysis was conducted on the keywords from 529 articles on siderophore secretion published between 2000 and 2024, drawn from the Web of Science Core Collection. The results of this analysis were visually represented through a knowledge map (Figure 6A), a keyword emergence view (Figure 6B), a dendrogram (Figure 6C), and a word cloud (Figure 6D). Prominent keywords such as “*Escherichia coli*”, “siderophore”, and “identification” were frequently observed, with their prominence highlighted by larger nodes in the visualizations.

The keyword emergence view, dendrogram, and word cloud consistently emphasize terms like “siderophore”, “iron”, and “*Escherichia coli*”. This repetition indicates a focused research interest in employing *E. coli* as a model organism for studying the synthesis and secretion of siderophores, alongside efforts to identify new siderophore compounds. Notable contributions include Mudhulkar et al. research on siderophores produced by halotolerant bacteria [92] and Oliveira et al.’s study on siderophore synthesis by Streptococcus mutans [93]. The analysis also highlighted these keywords’ significant betweenness centrality, reflecting their pivotal role in connecting various research themes within the field. A centrality value of ≥0.1 generally denotes substantial importance in the knowledge network, indicating these topics as central to current research discussions. This clustered keyword distribution reveals a strong emphasis on microbial synthesis and secretion of siderophores, particularly through studies on *E. coli* [94,95], with a lesser focus on siderophore activities in animals and plants. Furthermore, emerging research priorities include exploring the genes involved in the synthesis and secretion of siderophores [96,97,98] and investigating the potential toxicity of these compounds [99,100,101]. These areas represent crucial international research interests, likely driving future studies. The cluster analysis not only reaffirms the biochemical and microbial emphasis in siderophore research, but also uncovers potential literature gaps, particularly regarding non-microbial siderophore studies. These findings suggest directions for future research, advocating for a broader investigative scope incorporating novel biotechnological approaches to expand our understanding of siderophore dynamics across various biological systems. This strategic insight could lead to innovative applications and enhance our capability to exploit siderophores in environmental and medical fields.

### 3.3. Trends in Theme Evolution

**Figure 7:**
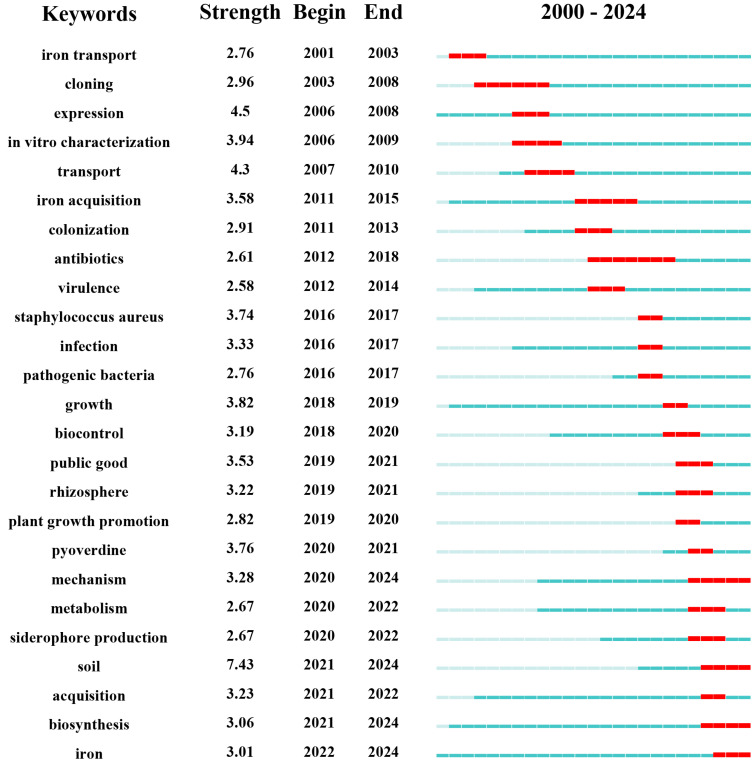
Spatial–temporal emergent analysis of core keywords in the field of iron carrier secretion from 2000 to 2024, made by Cite Space software, introduces the time and intensity of the emergence of keywords from 2000 to 2024. On the right side of the picture, the distribution range of keywords from 2000 to 2024 is intuitively displayed with red lines.

The evolution of research themes within the siderophore field over the past decades is evident from the analysis of keyword trends, as depicted in Figure 7. From 2000 to 2010, the primary focus was on terms like “iron transport”, “cloning”, “expression”, “in vitro characterization”, and “transport”. These keywords suggest a period dominated by studies on siderophore-producing bacteria, their secretion mechanisms, and the methods for their identification in laboratory settings.

Transitioning into the decade from 2011 to 2020, the research emphasis shifted toward keywords including “iron acquisition”, “colonization”, “antibiotics”, “virulence”, “Staphylococcus aureus”, “infection”, “pathogenic bacteria”, “growth”, “biocontrol”, “public good”, “rhizosphere”, “plant growth promotion”, “pyoverdine”, and “mechanism”. This shift indicates a broader focus on the roles of siderophores in pathogenesis, particularly with pathogens like *Staphylococcus aureus*, and their potential in biocontrol and plant growth enhancement, highlighting their ecological and agricultural applications. From 2021 to 2024, the research continued to evolve, concentrating on terms like “soil”, “acquisition”, “biosynthesis”, and “iron”, which underscore the environmental aspects of siderophore research. This recent focus reflects an enhanced interest in how siderophores interact with and impact environmental systems, particularly soil ecosystems.

**Figure 8:**
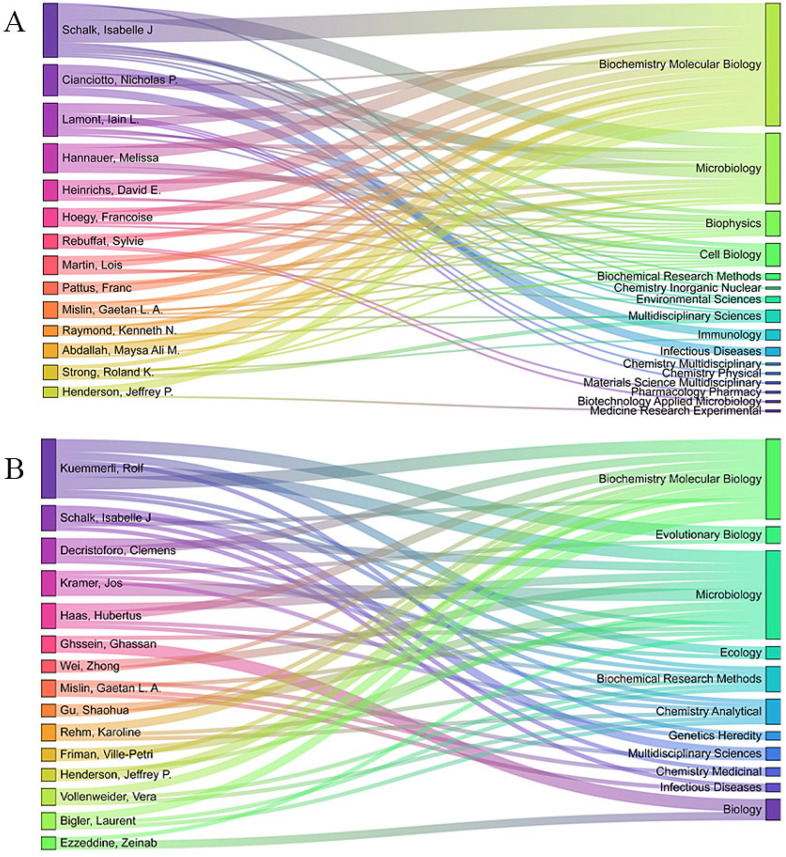
(**A**,**B**) Diagrams showing the relationship between the author of the generated web page and the publishing field. The left column is the author, the right column is the publishing field, and the connecting line in the middle represents the relationship between the author and the publishing field: (**A**) 2000 to 2017; and (**B**) 2018 to 2024.

The thematic evolution is further highlighted in Figure 8A,B, which show a significant expansion into interdisciplinary research areas since 2018. These figures illustrate the field’s diversification into evolutionary biology, ecology, chemical analytics, genetics, medical genetics, and biology. This interdisciplinary nature is likely driven by the increasing recognition of siderophores’ practical applications beyond traditional biochemical and microbial studies. The progression from initial laboratory-based studies focusing on production and secretion mechanisms to recent investigations into environmental impacts and interdisciplinary applications indicates a dynamic and expanding field. This evolution mirrors the growing understanding of siderophores’ complex roles in various biological and ecological processes. The thematic shifts reflect a broader scientific and practical interest in siderophores, underscoring their potential to address ecological challenges and enhance biological understanding across diverse systems.

## 4. Future Prospects

Following over two decades of intensive research, the field of siderophore secretion has yielded significant achievements and continues to delve deeper. Despite these advancements, several challenges persist: the mechanisms of siderophore secretion are not fully understood; research on siderophore synthesis and secretion in animals and plants is limited; the understanding of genes related to siderophore synthesis and their potential toxicity is insufficient; and comprehensive research into the impacts of siderophores on human health through food chains is lacking.

Considering these gaps, future research could beneficially focus on the following areas:

(1) Further Exploration of Siderophore Secretion Mechanisms: Deepen the understanding of the molecular mechanisms and regulatory pathways controlling siderophore production. This includes examining molecular interactions and the complex regulatory networks that influence siderophore production and secretion, aiming to uncover new details about these essential processes.
(2) Enhanced Study of Siderophores in Animals and Plants: Expand research into the roles and dynamics of siderophores in non-microbial organisms. By broadening the scope to include animal and plant systems, researchers can gain a holistic view of siderophore activities across various biological domains, potentially uncovering unique uses and functions.
(3) Investigation of Siderophore-Related Genes and Toxicity: Focus on identifying and characterizing the genes involved in siderophore synthesis and secretion, as well as assessing the toxicity of siderophores. This research could provide critical insights necessary for the safe and effective application of siderophores in agriculture, medicine, and environmental management.
(4) Development of New Techniques and Approaches: Pursue innovative research methods, utilizing advances in bioinformatics, genomics, and other cutting-edge technologies. This approach should also incorporate interdisciplinary collaboration, bringing together experts from chemistry, soil science, ecology, and other fields to foster a comprehensive and integrated understanding of siderophores. Such collaborative efforts can accelerate discoveries and applications of siderophores in various environmental and biological contexts.

These suggested research directions aim to fill the current knowledge gaps and catalyze transformative studies that could significantly enhance our understanding of siderophores’ ecological and biological roles. This strategic focus will likely lead to groundbreaking applications and insights, further establishing siderophores as crucial elements in biological systems and environmental health.

## Figures and Tables

**Table 1:** Iron carrier classification.

Type of Siderophore	Characteristic Functional Group	Characteristic	Siderophore-Producing Microorganism
Hydroxamic salt-type	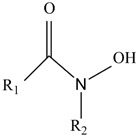	The most common in nature, the structure is more complex; it is more hydrophilic and prone to photooxidation	*Pseudomonas fluorescens* [26] *Aspergillus nidulans* [29]
Catechol salt-type	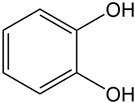	Strong lipophilicity and high affinity with Fe; strong resistance to environmental pH changes	*Escherichia coli* [28] *Klebsiella pneumoniae* [28]
Carboxylate-type	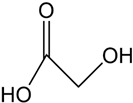	Potential ligands for the ocean iron cycle; some of them are photoactive	*Rhizobium meliloti* [30] *Staphylococcus aureus* [30]
Mixed-type	Mixed functional groups	It presents different characteristics according to different functional groups	*Rhodococcus erythropolis* [28] *Escherichia coli* [28]

**Table 2:** Examples of siderophores secreted by some plants.

Plant Species Name	Type of Siderophore	Year	References DOI
*Hordeum vulgare* L.	hydroxamate-type	2021	https://doi.org/10.1038/s41598-021-95736-7 [38]
*Hordeum vulgare* L.	catecholate-type	2021	https://doi.org/10.1080/00380768.2021.1947735 [39]
*Poaceae*	catecholate-type	2023	https://doi.org/10.1016/j.aca.2023.341718 [40]
*Poaceae*	catecholate-type	2023	https://doi.org/10.1002/jlcr.4064 [41]
*Polypogon monspenliensis*	unidentified	2020	https://doi.org/10.1007/s10265-020-01237-5 [37]

Note: “Unidentified” in the table means that the author has not given a precise type of siderophore in the corresponding article.

**Table 3:** Examples of siderophores secreted by some microorganisms.

Microbial Species Name	Type of Siderophore	Year	References DOI
*Escherichia vulneris*	hydroxamate-type	2020	https://doi.org/10.1016/j.jembe.2019.151290 [44]
*Enterobacter cancerogenus*	hydroxamate-type	2020	https://doi.org/10.1016/j.jembe.2019.151290 [44]
*Pantoea agglomerans*	hydroxamate-type	2020	https://doi.org/10.1016/j.jembe.2019.151290 [44]
*Enterobacter bugandensis*	hydroxamate-type	2020	https://doi.org/10.1016/j.jembe.2019.151290 [44]
*Erwinia amylovora* CFBP1430	hydroxamate-type	2022	https://doi.org/10.1128/aem.02433-21 [45]
*Burkholderia* sp. SX9	catecholate-type	2021	https://doi.org/10.1007/s11356-021-15996-8 [46]
*Myxobacterial Strain* MSr12020	catecholate-type	2022	https://doi.org/10.3390/microorganisms10101959 [47]
*Streptomyces tricolor Strain* HM10	carboxylate-type	2022	https://doi.org/10.3390/fermentation8080346 [24]
*Seudomonas aeruginosa* TonB	carboxylate-type	2023	https://doi.org/10.1002/1873-3468.14740 [23]
*Streptomyces morookaensiss*	carboxylate-type	2020	https://doi.org/10.1007/s40858-020-00396-z [48]

**Table 4:** Study on the secretion of siderophores by microorganisms from 2000 to 2023.

Years	Quantity
2000–2002	8
2003–2005	9
2006–2008	13
2009–2011	15
2012–2014	19
2015–2017	592
2018–2020	818
2021–2023	1024

## Data Availability

The data presented in this study are available on request from the corresponding author.
